# Phase Angle Measurement in Healthy Human Subjects through Bio-Impedance Analysis

**Published:** 2012

**Authors:** Satish Kumar, Aswini Dutt, Sandhya Hemraj, Shankar Bhat, Bhat Manipadybhima

**Affiliations:** 1 *Department of Physiology, Yenepoya Medical College, Deralakatte, Mangalore-575018. Karnataka, India*; 2 *Department of Radiology, Yenepoya Medical College, Deralakatte, Mangalore-575018. Karnataka, India*

**Keywords:** Bioelectrical Impedance, Body composition, Electric Resistance

## Abstract

**Objective(s):**

Bioelectrical impedance is the measure of impedance of the body. Impedance consists of electric resistance and reactance. Phase angle (PA) is the tan value of the ratio of reactance versus electric resistance. PA depends on cell membrane integrity and on body cell mass. There exists a correlation between PA values and body cell mass.

The objective of this study was to compare the PA values of normal individuals and their anthropometric measurements.

**Materials and Methods:**

Anthropometric measurements, Bioelectrical impedance analysis and PA measurements were done using Bodystat Quadscan 4000 machine on 42 healthy subjects between the age group of 18 to 50 yrs at a private hospital, Bangalore, Karnataka, India for eight months. Kolmogrov-Smirnov and Pearson’s correlation tests were used for data analysis.

**Results:**

The PA values were 7.321.17º in healthy subjects. PA values were significantly positively correlated with body mass index (BMI) (r= 0.011, *P*<0.001). The phase angle values for males and females were 7.43±0.98º and 7.05±1.1.58º, respectively.

**Conclusion:**

PA values positively correlated with BMI indicating the nutritional status of the study group. PA values were similar to the values to found in other studies.

## Introduction

Bioelectrical impedance is the measure of resistance and reactance of the body. It is used indirectly to measure the body fat composition. The total impedance is the total sum of impedance of different tissues. It was thought that if individual impedance is measured, then the different components of the body could be estimated (1). 

In the healthy living body, the cell membrane consists of a layer of non-conductive lipid material sandwiched between two layers of conductive protein molecules (1). The structure of cell membrane makes them a capacitive element which functions as capacitors when exposed to an alternating current. Theoretically, reactance is a measure of the volume of cell membrane capacitance and an indirect measure of the intracellular volume or body cell mass. Body fat, total body water and extra cellular water offer electric resistance to electrical current. Cell membranes and tissues interfaces offer capacitive reactance (2). 

Phase angle is a linear method of measuring the relationship between electric resistance (R) and reactance (R_c_) in series or parallel circuits. Taking the arc tangent value of the ratio of reactance versus electric resistance provides us with the phase angle value. Lower phase angles appear to be consistent with low reactance and equals either cell death or a breakdown in the selective permeability of the cell membrane. There is a significant difference in phase angle between healthy and disease states. The phase angle increases with improving clinical status (3-7). When repeated comparisons of body composition are required, BIA method can be as useful as Dexa scan (8). The difference can only be established if we have population reference values. 

***Concepts regarding physics and electrical nature of b*** ***ioelectrical impedance*** ***analysis***

Resistance: It is the opposition caused by the substance to the flow of current. It is the property of substance. Ohm’s law states that the resistance of a substance is proportional to the voltage drop of an applied current as it passes through a resistive substance.

Resistance (Ohms) = applied voltage drop/ current (amps)

The body is made up of both conductive and non-conductive tissues. The conducting tissues are lean tissues with large amount of water and conducting electrolytes. In nonconductive tissues like bone and fat, the fluid content and conducting electrolytes are low.

Reactance (Ohms) = 1/2 × π × frequency × Capacitance

Where reactance is in Ohms, frequency in Hertz, capacitance in Farads and π is a constant. In this equation, we can see that reactance is the reciprocal of frequency and capacitance. Therefore, reactance decreases as frequency increases. It means that reactance is virtually infinite at extremely low frequencies. The capacitance increases with large surface area of the plate, with distance between the two plates and the type of dielectric. Dielectric is a non-conductor. But in biological systems it is found that the smaller the quantity of the membranes, the greater the capacitance. This paradox is explained by the way the capacitors and resistors are connected in the body. It is connected both in series and parallel. Capacitance causes the administered current to lag behind the voltage and creates a phase shift, which is represented by the phase angle (1). The electrical impedance of the body is measured by introducing a small alternating current, into the body and measuring the potential difference that results.

A specific feature of any conductor is its critical frequency. It is the frequency at which the reactance is maximum. As frequency increases further, the applied current will penetrate into all the cell membranes. So the cell membrane loses its capacitive properties, hence the reactance falls. So the impedance will only be electric resistance (9, 10). It was found that 50 KHz was ideal to be used in humans to measure both electric resistance and maximum reactance. 

A study was conducted to investigate the prognostic role of PA in fifty-eight stage IV pancreatic carcinoma cases. This revealed that PA <5.0º had a median survival time of 6.3 (95% CI 3.5, 9.2) months (n 29), while those with PA >5.0º had a median survival time of 10.2 (95% CI 9.6, 10.8) months (n 29); this difference was statistically significant (*P*=0.02) (11). 

Phase angle normal values range from 8-15º at 50 KHz in healthy Malawian adults (12). Mean phase angle for males, females and overall were 7.48±1.1º, 6.53±1.01º and 6.93±1.15º respectively. Whites were having 7.00±1.01º, Asians had 6.55±1.10º, African Americans had 7.21±1.19º, Hispanics had 7.33±1.13º and the rest had 7.45±0.48º (13).

The phase angle values have been studied in different countries across various populations (12, 13). The present study was designed to analyze the phase angle values for the normal Indian individuals and their anthropometric measurements.

## Materials and Methods

Forty-two healthy subjects were chosen from the student community and general population in the age group of 18-50 yrs at a private hospital, Bangalore, Karnataka, India. Thirty subjects were males and twelve were females. Ethical clearance was obtained from Institute’s Ethical clearance review board. Informed consent was obtained using a specially designed consent form. Subjects were weighed in clothing using a digital load cell balance (Soehnle, West Germany) which had a precision of 0.1 kg. The heights of the subjects were recorded without footwear, using a vertically mobile scale (Holtain, Crymych, United Kingdom) and expressed to the nearest 0.1 cm. Body mass index (BMI) was calculated from the height and weight as follows; BMI= weight (kg)/height^2^ (meters). 

***Exclusion*** ***criteria***

- History of any chronic illness
- Alcohol - more than 2 standard drinks per day

- Signs of dehydration
- Any implants
- Fever
- Menstruation and pregnancy

***Phase angle measurement protocol***

Bioelectrical impedance analysis is performed with the Quad scan instrument (BODYSTAT Quadscan 4000), applying a current of 500 to 800 micro amperes and 50 KHz frequency. After an overnight fast, the subjects were made to lie down supine on a bed in the metabolic lab for 10 min. History of any pacemaker or orthopedic hardware implant was taken along with history of food intake and recent exercise. The subjects were asked to separate the legs for 30º to 40º. Any jewellery on the person was removed. Ordinary ECG electrodes were used under aseptic conditions. Electrodes were applied on the right side with injecting electrodes placed on dorsum of hand and feet on the metacarpal and metatarsals respectively. The reading electrodes were placed between the medial and lateral malleolus of the same side. The reading electrodes of the wrist were placed between radial styloid and ulnar prominence of the wrist. The distance between injecting and reading electrodes was 5 cm. 

The subjects were asked not to move when the instrument was measuring the Bioelectrical impedance. Single measurement was taken. In case of erroneous readings, the electrodes were re-applied and the measurement was repeated. Phase angle was calculated using the phase angle calculation software version 1.0 using the impedance at 50 KHz. 

## Results

**Figure 1:**
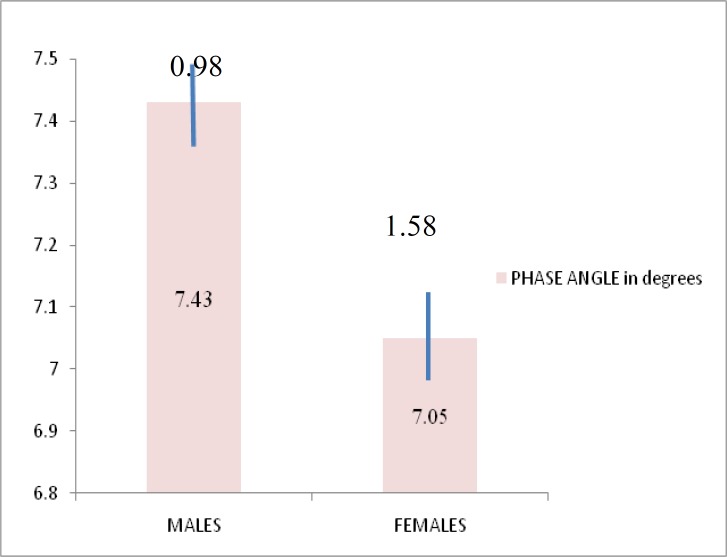
Phase angle and its standard deviation in males and females

**Figure 2:**
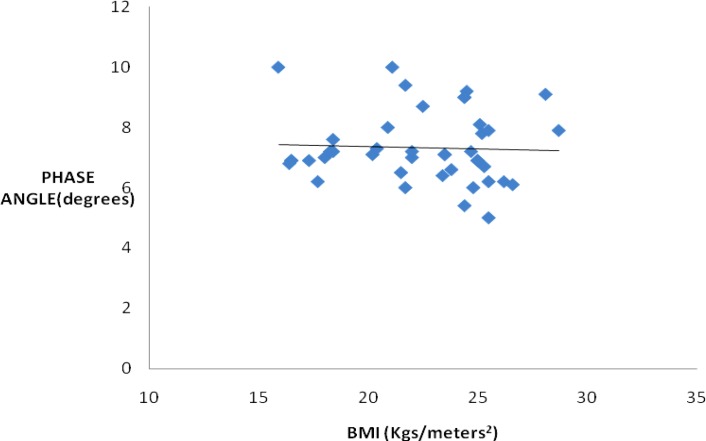
Correlation between phase angle and BMI

 The data was analysed statistically for normal distribution. Microsoft Excel and SPSS version 16.0, Kolmogrov-Smirnov and Pearson’s correlation tests were used for data analysis. Table 1 shows age in yrs, anthropometric measurements of the study group, impedance at 50 KHz and PA values. PA values for males and females are shown in Figure 1. Results revealed that PA values were significantly correlated with BMI in the study group (r=0.011, *P*<0.001) (Figure 2). 

**Table 1:** Anthropometric measurements of study group

Parameters	Mean ± SD*	Range
Age (yrs)	32.64±12.25	18-50
Height (cm)	164.26±8.0	148.6-184
Weight (Kg)	60.47±10.14	41.3-79.2
BMI	22.39±3.42	15.9-28.7
Impedance at 50 K Hz (Ohm)	594.16±73.71	489-740
Phase angle (degree)	7.32±1.17	5-10

*SD Standard Deviation

*SD Standard Deviation

## Discussion

This study has demonstrated that the range of phase angle values in healthy subjects is 7.32±1.17º. According to the studies of Cristina *et al* the population reference values of phase angles of Asians and overall population were 6.55±1.10º 6.93±1.15º respectively (13). Bioelectrical impedance analysis (BIA) was found to be as good as anthropometry in predicting body composition (14). Norman *et al* have mentioned the benefits of bio-impedance when calculation of body composition is not feasible (15). 

One of the advantages of BIA over BMI alone is that BIA can differentiate the degree of body fat and body cell mass in individuals. It also can be used to assess the prognosis of chronic diseases like HIV infection, chronic obstructive pulmonary disease (16), tuberculosis, pancreatic cancer and colorectal cancer (4, 12). Malnutrition and inflammation have a strong impact on PA in diseased individuals (17). BIA is a safe, cheap and easy method of measuring body composition. 

But the disadvantage is that it has to be measured under ideal settings where room temperature, exercise, electrode placement and food intake are controlled (18). Changes in the hydration status can alter the impedance (19). Regarding the safety, perception depends on the intensity and threshold of the stimulus (much lower than 800 micro amperes), more in males and similar in females and children (20, 21). It is not validated in altered hydration status. In the case of pregnant women and children, risks are not evaluated. 

## Conclusions

This study showed the normal values of the phase angle in both genders which can be used to be compared with diseased states. Phase angle measurement in larger Indian population in different age groups needs to be studied further.
